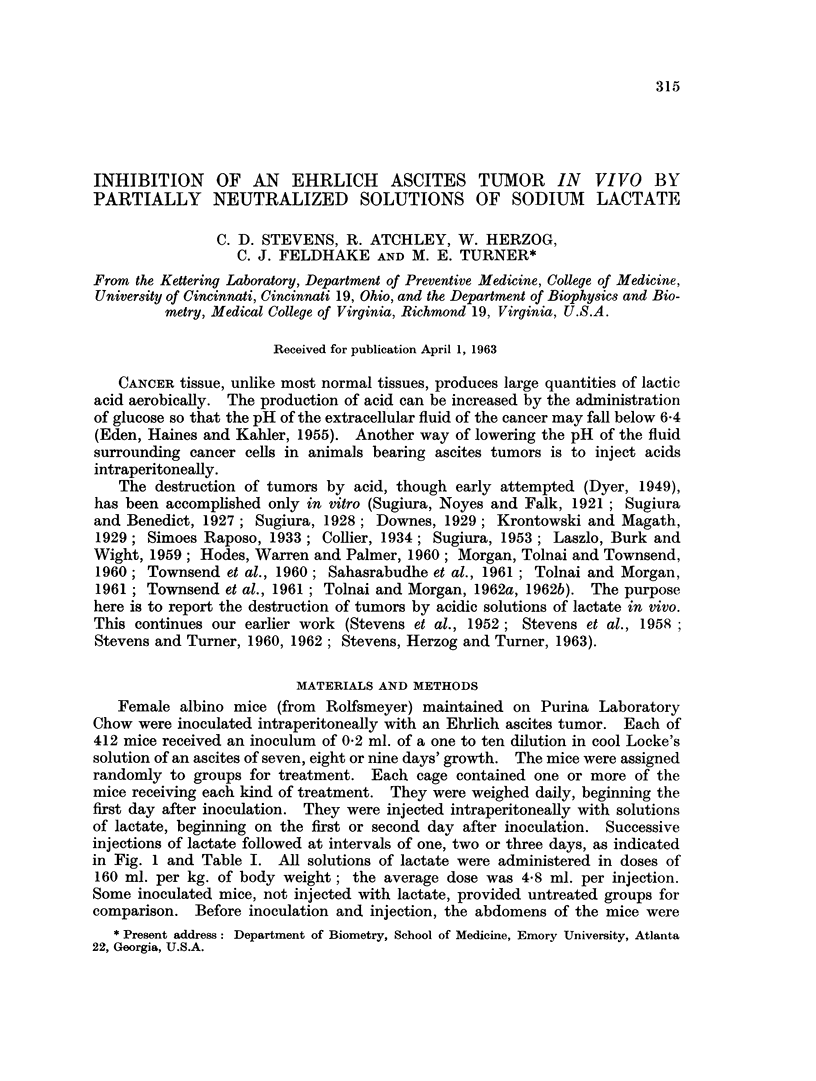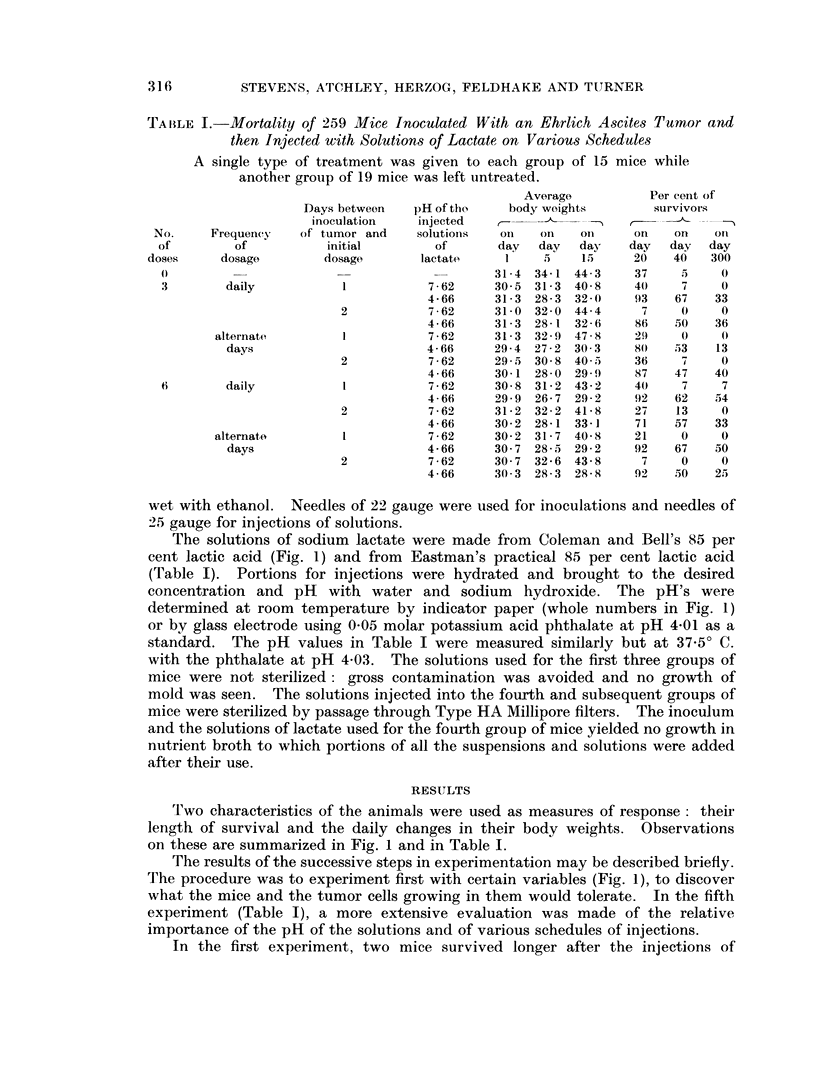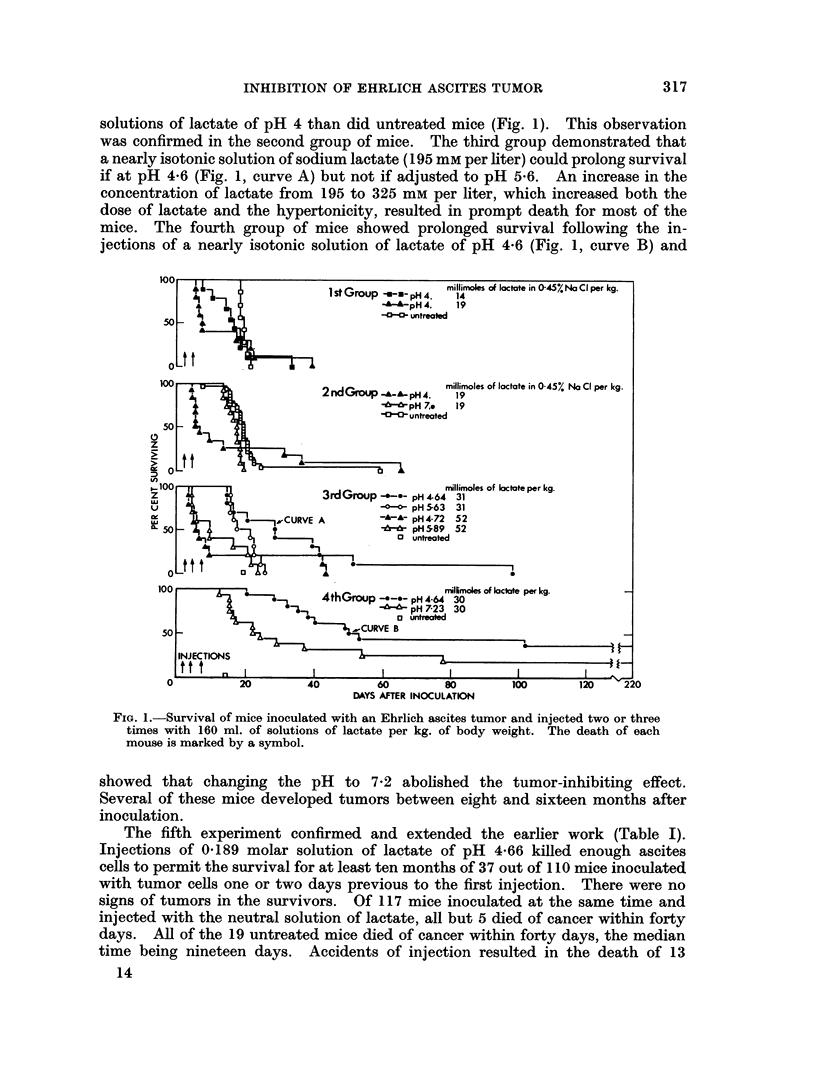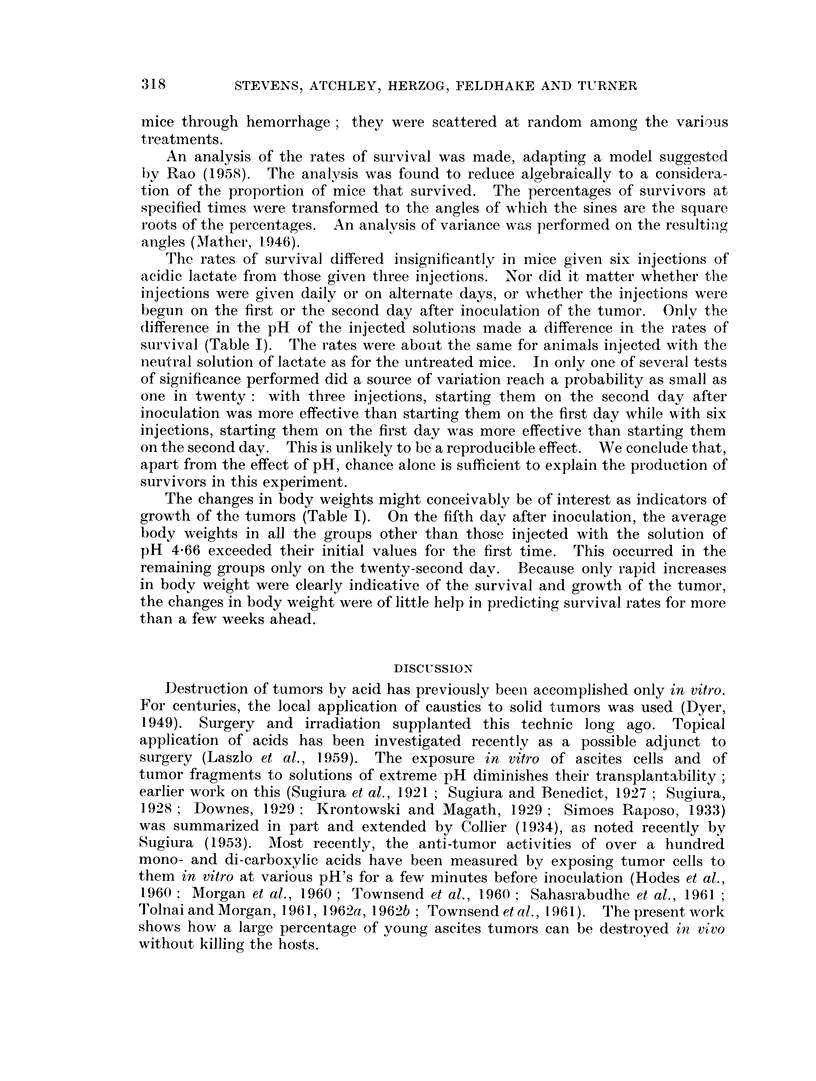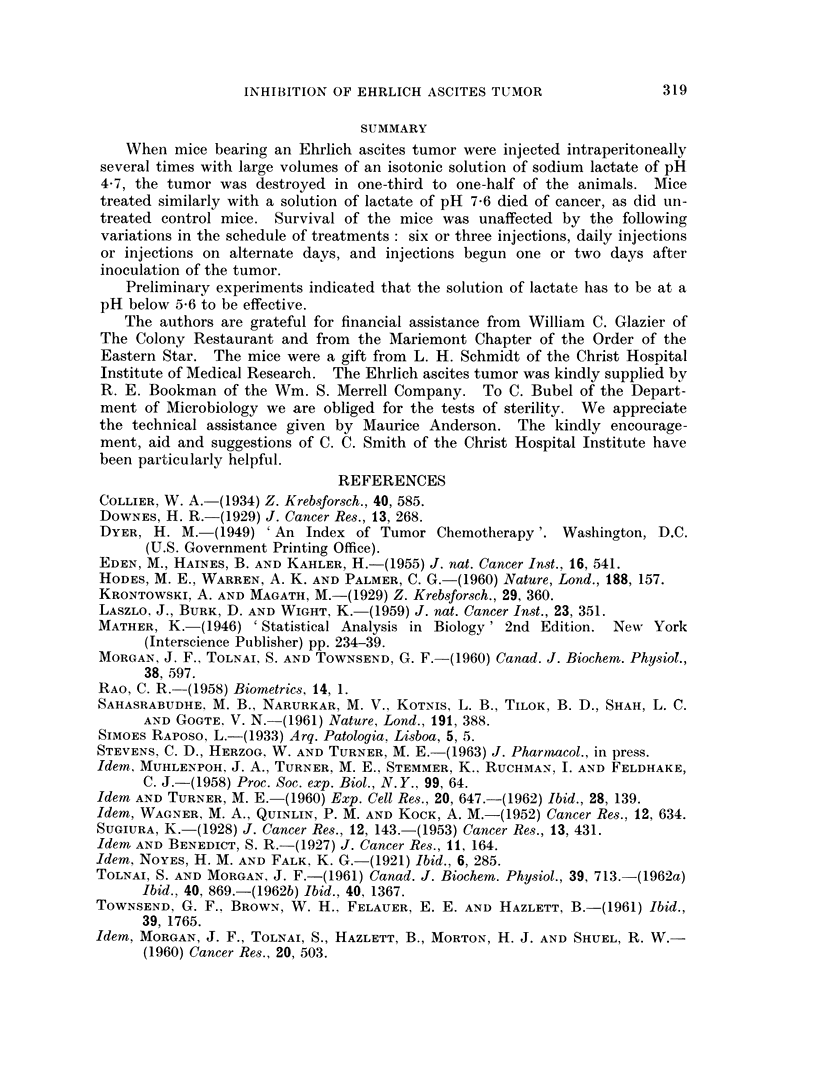# Inhibition of an Ehrlich Ascites Tumor In Vivo by Partially Neutralized Solutions of Sodium Lactate

**DOI:** 10.1038/bjc.1963.45

**Published:** 1963-06

**Authors:** C. D. Stevens, R. Atchley, W. Herzog, C. J. Feldhake, M. E. Turner


### INHIBITION OF AN EHRLICH ASCITES TUMOR *IN VIVO* BY PARTIALLY NEUTRALIZED SOLUTIONS OF SODIUM LACTATE

C. D. STEVENS, R. ATCHLEY, W. HERZOG,
C. J. FELDHAKE AND M. E. TURNER*

*From the Kettering Laboratory, Department of Preventive Medicine, College of Medicine, University of Cincinnati, Cincinnati* 19, *Ohio, and the Department of Biophysics and Biometry, Medical College of Virginia, Richmond* 19, *Virginia, U.S.A.*

Received for publication April 1, 1963

CANCER tissue, unlike most normal tissues, produces large quantities of lactic acid aerobically. The production of acid can be increased by the administration of glucose so that the pH of the extracellular fluid of the cancer may fall below 6·4 (Eden, Haines and Kahler, 1955). Another way of lowering the pH of the fluid surrounding cancer cells in animals bearing ascites tumors is to inject acids intraperitoneally.

The destruction of tumors by acid, though early attempted (Dyer, 1949), has been accomplished only *in vitro* (Sugiura, Noyes and Falk, 1921 ; Sugiura and Benedict, 1927 ; Sugiura, 1928 ; Downes, 1929 ; Krontowski and Magath, 1929 ; Simoes Raposo, 1933 ; Collier, 1934 ; Sugiura, 1953 ; Laszlo, Burk and Wight, 1959 ; Hodes, Warren and Palmer, 1960 ; Morgan, Tolnai and Townsend, 1960 ; Townsend *et al.*, 1960 ; Sahasrabudhe *et al.*, 1961 ; Tolnai and Morgan, 1961 ; Townsend *et al.*, 1961 ; Tolnai and Morgan, 1962*a*, 1962*b*). The purpose here is to report the destruction of tumors by acidic solutions of lactate *in vivo*. This continues our earlier work (Stevens *et al.*, 1952 ; Stevens *et al.*, 1958 ; Stevens and Turner, 1960, 1962 ; Stevens, Herzog and Turner, 1963).

### MATERIALS AND METHODS

Female albino mice (from Rolfsmeyer) maintained on Purina Laboratory Chow were inoculated intraperitoneally with an Ehrlich ascites tumor. Each of 412 mice received an inoculum of 0·2 ml. of a one to ten dilution in cool Locke's solution of an ascites of seven, eight or nine days' growth. The mice were assigned randomly to groups for treatment. Each cage contained one or more of the mice receiving each kind of treatment. They were weighed daily, beginning the first day after inoculation. They were injected intraperitoneally with solutions of lactate, beginning on the first or second day after inoculation. Successive injections of lactate followed at intervals of one, two or three days, as indicated in Fig. 1 and Table I. All solutions of lactate were administered in doses of 160 ml. per kg. of body weight ; the average dose was 4·8 ml. per injection. Some inoculated mice, not injected with lactate, provided untreated groups for comparison. Before inoculation and injection, the abdomens of the mice were

* Present address : Department of Biometry, School of Medicine, Emory University, Atlanta 22, Georgia, U.S.A.

TABLE I.—*Mortality of 259 Mice Inoculated With an Ehrlich Ascites Tumor and then Injected with Solutions of Lactate on Various Schedules*

A single type of treatment was given to each group of 15 mice while another group of 19 mice was left untreated.

| No. of doses | Frequency of dosage | Days between inoculation of tumor and initial dosage | pH of the injected solutions of lactate | Average body weights on day 1 | on day 5 | on day 15 | Per cent of survivors on day 20 | on day 40 | on day 300 |
|---|---|---|---|---|---|---|---|---|---|
| 0 | — | — | — | 31·4 | 34·1 | 44·3 | 37 | 5 | 0 |
| 3 | daily | 1 | 7·62 | 30·5 | 31·3 | 40·8 | 40 | 7 | 0 |
| | | | 4·66 | 31·3 | 28·3 | 32·0 | 93 | 67 | 33 |
| | | 2 | 7·62 | 31·0 | 32·0 | 44·4 | 7 | 0 | 0 |
| | | | 4·66 | 31·3 | 28·1 | 32·6 | 86 | 50 | 36 |
| | alternate days | 1 | 7·62 | 31·3 | 32·9 | 47·8 | 29 | 0 | 0 |
| | | | 4·66 | 29·4 | 27·2 | 30·3 | 80 | 53 | 13 |
| | | 2 | 7·62 | 29·5 | 30·8 | 40·5 | 36 | 7 | 0 |
| | | | 4·66 | 30·1 | 28·0 | 29·9 | 87 | 47 | 40 |
| 6 | daily | 1 | 7·62 | 30·8 | 31·2 | 43·2 | 40 | 7 | 7 |
| | | | 4·66 | 29·9 | 26·7 | 29·2 | 92 | 62 | 54 |
| | | 2 | 7·62 | 31·2 | 32·2 | 41·8 | 27 | 13 | 0 |
| | | | 4·66 | 30·2 | 28·1 | 33·1 | 71 | 57 | 33 |
| | alternate days | 1 | 7·62 | 30·2 | 31·7 | 40·8 | 21 | 0 | 0 |
| | | | 4·66 | 30·7 | 28·5 | 29·2 | 92 | 67 | 50 |
| | | 2 | 7·62 | 30·7 | 32·6 | 43·8 | 7 | 0 | 0 |
| | | | 4·66 | 30·3 | 28·3 | 28·8 | 92 | 50 | 25 |

wet with ethanol. Needles of 22 gauge were used for inoculations and needles of 25 gauge for injections of solutions.

The solutions of sodium lactate were made from Coleman and Bell's 85 per cent lactic acid (Fig. 1) and from Eastman's practical 85 per cent lactic acid (Table I). Portions for injections were hydrated and brought to the desired concentration and pH with water and sodium hydroxide. The pH's were determined at room temperature by indicator paper (whole numbers in Fig. 1) or by glass electrode using 0·05 molar potassium acid phthalate at pH 4·01 as a standard. The pH values in Table I were measured similarly but at 37·5° C. with the phthalate at pH 4·03. The solutions used for the first three groups of mice were not sterilized : gross contamination was avoided and no growth of mold was seen. The solutions injected into the fourth and subsequent groups of mice were sterilized by passage through Type HA Millipore filters. The inoculum and the solutions of lactate used for the fourth group of mice yielded no growth in nutrient broth to which portions of all the suspensions and solutions were added after their use.

### RESULTS

Two characteristics of the animals were used as measures of response : their length of survival and the daily changes in their body weights. Observations on these are summarized in Fig. 1 and in Table I.

The results of the successive steps in experimentation may be described briefly. The procedure was to experiment first with certain variables (Fig. 1), to discover what the mice and the tumor cells growing in them would tolerate. In the fifth experiment (Table I), a more extensive evaluation was made of the relative importance of the pH of the solutions and of various schedules of injections.

In the first experiment, two mice survived longer after the injections of solutions of lactate of pH 4 than did untreated mice (Fig. 1). This observation was confirmed in the second group of mice. The third group demonstrated that a nearly isotonic solution of sodium lactate (195 mM per liter) could prolong survival if at pH 4·6 (Fig. 1, curve A) but not if adjusted to pH 5·6. An increase in the concentration of lactate from 195 to 325 mM per liter, which increased both the dose of lactate and the hypertonicity, resulted in prompt death for most of the mice. The fourth group of mice showed prolonged survival following the injections of a nearly isotonic solution of lactate of pH 4·6 (Fig. 1, curve B) and

Fig. 1.—Survival of mice inoculated with an Ehrlich ascites tumor and injected two or three times with 160 ml. of solutions of lactate per kg. of body weight. The death of each mouse is marked by a symbol.

showed that changing the pH to 7·2 abolished the tumor-inhibiting effect. Several of these mice developed tumors between eight and sixteen months after inoculation.

The fifth experiment confirmed and extended the earlier work (Table I). Injections of 0·189 molar solution of lactate of pH 4·66 killed enough ascites cells to permit the survival for at least ten months of 37 out of 110 mice inoculated with tumor cells one or two days previous to the first injection. There were no signs of tumors in the survivors. Of 117 mice inoculated at the same time and injected with the neutral solution of lactate, all but 5 died of cancer within forty days. All of the 19 untreated mice died of cancer within forty days, the median time being nineteen days. Accidents of injection resulted in the death of 13

mice through hemorrhage ; they were scattered at random among the various treatments.

An analysis of the rates of survival was made, adapting a model suggested by Rao (1958). The analysis was found to reduce algebraically to a consideration of the proportion of mice that survived. The percentages of survivors at specified times were transformed to the angles of which the sines are the square roots of the percentages. An analysis of variance was performed on the resulting angles (Mather, 1946).

The rates of survival differed insignificantly in mice given six injections of acidic lactate from those given three injections. Nor did it matter whether the injections were given daily or on alternate days, or whether the injections were begun on the first or the second day after inoculation of the tumor. Only the difference in the pH of the injected solutions made a difference in the rates of survival (Table I). The rates were about the same for animals injected with the neutral solution of lactate as for the untreated mice. In only one of several tests of significance performed did a source of variation reach a probability as small as one in twenty : with three injections, starting them on the second day after inoculation was more effective than starting them on the first day while with six injections, starting them on the first day was more effective than starting them on the second day. This is unlikely to be a reproducible effect. We conclude that, apart from the effect of pH, chance alone is sufficient to explain the production of survivors in this experiment.

The changes in body weights might conceivably be of interest as indicators of growth of the tumors (Table I). On the fifth day after inoculation, the average body weights in all the groups other than those injected with the solution of pH 4·66 exceeded their initial values for the first time. This occurred in the remaining groups only on the twenty-second day. Because only rapid increases in body weight were clearly indicative of the survival and growth of the tumor, the changes in body weight were of little help in predicting survival rates for more than a few weeks ahead.

### DISCUSSION

Destruction of tumors by acid has previously been accomplished only *in vitro*. For centuries, the local application of caustics to solid tumors was used (Dyer, 1949). Surgery and irradiation supplanted this technic long ago. Topical application of acids has been investigated recently as a possible adjunct to surgery (Laszlo *et al.*, 1959). The exposure *in vitro* of ascites cells and of tumor fragments to solutions of extreme pH diminishes their transplantability ; earlier work on this (Sugiura *et al.*, 1921 ; Sugiura and Benedict, 1927 ; Sugiura, 1928 ; Downes, 1929 ; Krontowski and Magath, 1929 ; Simoes Raposo, 1933) was summarized in part and extended by Collier (1934), as noted recently by Sugiura (1953). Most recently, the anti-tumor activities of over a hundred mono- and di-carboxylic acids have been measured by exposing tumor cells to them *in vitro* at various pH's for a few minutes before inoculation (Hodes *et al.*, 1960 ; Morgan *et al.*, 1960 ; Townsend *et al.*, 1960 ; Sahasrabudhe *et al.*, 1961 ; Tolnai and Morgan, 1961, 1962*a*, 1962*b* ; Townsend *et al.*, 1961). The present work shows how a large percentage of young ascites tumors can be destroyed *in vivo* without killing the hosts.

SUMMARY

When mice bearing an Ehrlich ascites tumor were injected intraperitoneally several times with large volumes of an isotonic solution of sodium lactate of pH 4·7, the tumor was destroyed in one-third to one-half of the animals. Mice treated similarly with a solution of lactate of pH 7·6 died of cancer, as did untreated control mice. Survival of the mice was unaffected by the following variations in the schedule of treatments : six or three injections, daily injections or injections on alternate days, and injections begun one or two days after inoculation of the tumor.

Preliminary experiments indicated that the solution of lactate has to be at a pH below 5·6 to be effective.

The authors are grateful for financial assistance from William C. Glazier of The Colony Restaurant and from the Mariemont Chapter of the Order of the Eastern Star. The mice were a gift from L. H. Schmidt of the Christ Hospital Institute of Medical Research. The Ehrlich ascites tumor was kindly supplied by R. E. Bookman of the Wm. S. Merrell Company. To C. Bubel of the Department of Microbiology we are obliged for the tests of sterility. We appreciate the technical assistance given by Maurice Anderson. The kindly encouragement, aid and suggestions of C. C. Smith of the Christ Hospital Institute have been particularly helpful.

REFERENCES

COLLIER, W. A.—(1934) *Z. Krebsforsch.*, **40**, 585.

DOWNES, H. R.—(1929) *J. Cancer Res.*, **13**, 268.

DYER, H. M.—(1949) 'An Index of Tumor Chemotherapy'. Washington, D.C. (U.S. Government Printing Office).

EDEN, M., HAINES, B. AND KAHLER, H.—(1955) *J. nat. Cancer Inst.*, **16**, 541.

HODES, M. E., WARREN, A. K. AND PALMER, C. G.—(1960) *Nature, Lond.*, **188**, 157.

KRONTOWSKI, A. AND MAGATH, M.—(1929) *Z. Krebsforsch.*, **29**, 360.

LASZLO, J., BURK, D. AND WIGHT, K.—(1959) *J. nat. Cancer Inst.*, **23**, 351.

MATHER, K.—(1946) 'Statistical Analysis in Biology' 2nd Edition. New York (Interscience Publisher) pp. 234–39.

MORGAN, J. F., TOLNAI, S. AND TOWNSEND, G. F.—(1960) *Canad. J. Biochem. Physiol.*, **38**, 597.

RAO, C. R.—(1958) *Biometrics*, **14**, 1.

SAHASRABUDHE, M. B., NARURKAR, M. V., KOTNIS, L. B., TILOK, B. D., SHAH, L. C. AND GOGTE, V. N.—(1961) *Nature, Lond.*, **191**, 388.

SIMOES RAPOSO, L.—(1933) *Arq. Patologia, Lisboa*, **5**, 5.

STEVENS, C. D., HERZOG, W. AND TURNER, M. E.—(1963) *J. Pharmacol.*, in press.

*Idem*, MUHLENPOH, J. A., TURNER, M. E., STEMMER, K., RUCHMAN, I. AND FELDHAKE, C. J.—(1958) *Proc. Soc. exp. Biol., N.Y.*, **99**, 64.

*Idem* AND TURNER, M. E.—(1960) *Exp. Cell Res.*, **20**, 647.—(1962) *Ibid.*, **28**, 139.

*Idem*, WAGNER, M. A., QUINLIN, P. M. AND KOCK, A. M.—(1952) *Cancer Res.*, **12**, 634.

SUGIURA, K.—(1928) *J. Cancer Res.*, **12**, 143.—(1953) *Cancer Res.*, **13**, 431.

*Idem* AND BENEDICT, S. R.—(1927) *J. Cancer Res.*, **11**, 164.

*Idem*, NOYES, H. M. AND FALK, K. G.—(1921) *Ibid.*, **6**, 285.

TOLNAI, S. AND MORGAN, J. F.—(1961) *Canad. J. Biochem. Physiol.*, **39**, 713.—(1962*a*) *Ibid.*, **40**, 869.—(1962*b*) *Ibid.*, **40**, 1367.

TOWNSEND, G. F., BROWN, W. H., FELAUER, E. E. AND HAZLETT, B.—(1961) *Ibid.*, **39**, 1765.

*Idem*, MORGAN, J. F., TOLNAI, S., HAZLETT, B., MORTON, H. J. AND SHUEL, R. W.—(1960) *Cancer Res.*, **20**, 503.